# Estimation of Zika virus prevalence by appearance of microcephaly

**DOI:** 10.1186/s12879-016-2076-z

**Published:** 2016-12-12

**Authors:** C. M. Saad-Roy, P. van den Driessche, Junling Ma

**Affiliations:** Department of Mathematics and Statistics, University of Victoria, Victoria, BC V8W 2Y2 Canada

**Keywords:** Zika prevalence, Microcephaly, Vector transmission, Sexual transmission, Mathematical model, Contact network

## Abstract

**Background:**

There currently is a severe Zika Virus (ZIKV) epidemic in Brazil and other South American countries. Due to international travel, this poses severe public health risk of ZIKV importation to other countries. We estimate the prevalence of ZIKV in an import region by the time a microcephaly case is detected, since microcephaly is presently the most significant indication of ZIKV presence.

**Methods:**

We establish a mathematical model to describe ZIKV spread from a source region to an import region. This model incorporates both vector transmission (between humans and mosquitoes) and sexual transmission (from males to females). We take account of population structure through a contact network for sexually active individuals. Parameter values of our model are either taken from the literature or estimated from travel data.

**Results:**

This model gives us the probability distribution of time until detection of the first microcephaly case. Based on current field observations, our results also indicate that the percentage of infected pregnant women that results in fetal abnormalities is more likely to be on the smaller end of the 1%–30% spectrum that is currently hypothesized. Our model predicts that for import regions with at least 250,000 people, on average 1,000–12,000 will have been infected by the time of the first detection of microcephaly, and on average 200–1,500 will be infectious at this time. Larger population sizes do not significantly change our predictions.

**Conclusions:**

By the first detection of a microcephaly case, a sizable fraction of the population will have been infected by ZIKV. It is thus clear that adequate surveillance, isolation, and quarantine are needed in susceptible import regions to stop the dissemination of a Zika epidemic.

**Electronic supplementary material:**

The online version of this article (doi:10.1186/s12879-016-2076-z) contains supplementary material, which is available to authorized users.

## Background

In March 2015, an outbreak of Zika virus (hereafter referred to as ZIKV) was discovered in Bahia, Brazil [1]. Recent findings [2] attribute the introduction of ZIKV in Brazil to a single imported infection, estimated to be during the latter half of 2013. This outbreak is currently ongoing with public health efforts to mitigate the dispersal of ZIKV throughout other populations. Recent ZIKV outbreaks before the current outbreak in Brazil include outbreaks in Pacific Islands, such as in 2007 in Yap Island, Micronesia [3] and in 2013 in French Polynesia [4]. Currently, there are two main strains of ZIKV, namely an African lineage and an Asian lineage [5].

Acute ZIKV infections in humans may result in fever, macopapular rash, conjunctivitis, muscle pain and joint aches [6]. These symptoms of ZIKV infections are usually mild and difficult to differentiate from other viral infections thus resulting in vast underreporting of ZIKV cases. However, epidemiological studies of recent outbreaks of ZIKV indicate associations between ZIKV and neurological disorders, especially microcephaly and significant brain anomalies in fetuses of infected pregnant females [7]. This has been confirmed during both the French Polynesia outbreak [8] and the current outbreak in Brazil [9]; see [10] and [11] for specific cases of fetal abnormalities during infected pregnancies. In addition, both the infection by the Brazilian strain [12] and the infection by the SZ01 Asian strain [13] have been demonstrated to cause microcephaly in mice fetuses.

For a population that is not under active surveillance, an abnormal fetus is very likely the first indication of a ZIKV epidemic in the population. However, starting from the importation of ZIKV into a population, it may take some time for ZIKV to infect a pregnant female due to the low fraction of such females in the population (approximately 1% in the United States [14]). Moreover, only infections in their first trimester are more likely to result in an increased risk for fetal abnormality such as microcephaly [8], and such an abnormal fetus can be detected at roughly 20 weeks [11]. Thus, by the time a microcephaly case is detected, ZIKV could have spread in the population for a long time, and caused an epidemic. Estimates of the final size of ZIKV epidemics on Yap Island and in French Polynesia are 73% and 66%, respectively [3, 8], showing that a significant proportion of individuals in a susceptible population become infected with ZIKV during an outbreak. In conjunction with the association of ZIKV with neurological disorders, these percentages indicate potential repercussions on a large scale.

In this paper, we quantify the prevalence and total infections of ZIKV in a region (e.g., a city, a state or a country) at the time of the first detection of microcephaly, estimates that are crucial for public health planning at the start of a ZIKV outbreak.

## Methods

We develop a mathematical model for the spread of ZIKV, which is transmitted to humans by mosquitoes of the *Aedes* genus; for e.g., *A. aegypti*, *A. albopictus* and *A. africanus* mosquitoes [15]. These mosquitoes also transmit yellow fever, dengue and chikungunya. Mathematical models for these vector transmitted diseases have been extensively studied [16, 17]. However, ZIKV can also be transmitted through sexual contact from an infected male to a susceptible female [15, 18–20]. In fact, ZIKV persists in semen much longer than in blood and other bodily fluids [21]. Thus vector transmission may have a shorter infectious period than sexual transmission. Recovery after infection seems to confer long-term immunity for individuals [22]. There currently exist no cure, no treatment, and no vaccine for ZIKV. This effectively renders every individual that has not contracted ZIKV a susceptible one, and indicates a large potential for the dissemination of ZIKV through human populations.

**Fig. 1:**
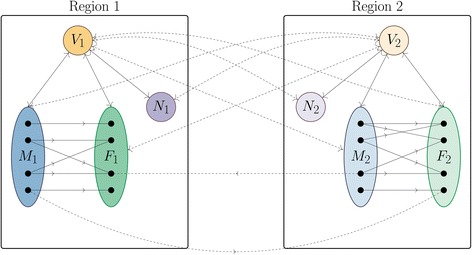
Flowchart of two-region model for ZIKV transmission with vectors *V*, males *M*, females *F*, sexually inactive humans *N*, and subscripts representing regions. The solid lines represent contacts within a region, and dashed curves represent contacts between regions

Our mathematical model considers two regions to incorporate the importation of cases from a “source” region (Region 1) experiencing an epidemic into a completely susceptible “import” region (Region 2). Figure 1 illustrates our model for the transmission of ZIKV in two regions, and Table 1 summarizes parameter values and their ranges; see Additional file 1: for justification of the model and of parameter values.

**Table 1:** Parameter definitions, values and ranges for two-region model

Parameter	Definition (time^-1^)	Estimated baseline value (day^-1^)	Range (day^-1^)	Source
${\beta}_V{{{}_{{}_1}}_{H_1}}_{,\kern0.5em }{\beta}_{V_2{H}_2}$	Transmission rate, mosquitoes to humans within region	0.105	0.0975 to 0.1125	[17]
${\beta}_H{{{}_{{}_1}}_{V_1}}_{,\kern0.5em }{\beta}_{H_2{V}_2}$	Transmission rate, humans to mosquitoes within region	0.105	0.0975 to 0.1125	[17]
${\beta}_V{{{}_{{}_1}}_{H_2}}_{,\kern0.5em }{\beta}_{V_2{H}_1}$	Transmission rate, mosquitoes to humans between regions	0.00003	0.00003 to 0.00006	Estimated (TA, Additional file 1), [17, 29]
${\beta}_H{{{}_{{}_1}}_{V_2}}_{,\kern0.5em }{\beta}_{H_2{V}_1}$	Transmission rate, humans to mosquitoes between regions	0.00003	0.00003 to 0.00006	Estimated (TA, Additional file 1), [17, 29]
*1/ γ*_*B*_	Mean infectious period from blood	7	4 to 11	[30]
*1/ γ*_*B*_ *+ 1/ γ*_*B*_	Mean infectious period from semen	62	57 to 67	[21, 30]
${\beta}_{M_1{F}_1,\kern0.5em }{\beta}_{M_2{F}_2}$	Transmission rate, males to females within region	0.5	0.3 to 0.7	Estimated
${\beta}_{M_1{F}_2,\kern0.5em }{\beta}_{M_2{F}_1}$	Transmission rate, males to females between region	0.01	0.005-0.105	Estimated
*d*	Death rate of mosquitoes	1/50		[31]

The time to the first infection *T*_*p*_ of a pregnant female in Region 2 can be estimated from this model. New cases per day in pregnant females that result in microcephaly can be modeled as a non-homogeneous Poisson process with rate

*λ*(*t*) = − *p* × *z* × (rate of change of susceptible sexually active females in Region 2),

where *p* is the proportion of pregnant females in the sexually active female population and *z* is the proportion of pregnancies that are affected by ZIKV and result in fetal abnormalities. The probability density function for *T*_*p*_ is *λ*(*t*)*exp(*∫_o_^*t*^ *λ*(*τ*)*dτ)*. For example, 1% of the US population are pregnant females [14], which corresponds to *p* = 3% since 1*/*3 of the population is sexually active females. It is estimated that *z* = 29% in Brazil [9], whereas it is estimated that *z* = 1% based on data from the ZIKV outbreak in French Polynesia [8].

The time to the first detection of a microcephaly case *T*_*d*_ can then be computed by adding the delay from the infection of a pregnant female to the detection of abnormality in the fetus. Fetal abnormality most likely results from ZIKV infection in the first trimester, as shown in the 2013-2014 outbreak of ZIKV in French Polynesia [8]. Because the infection of the mother can occur any time during her pregnancy, by the detection at 20 weeks, i.e., 140 days [11], *T*_*d*_ = *T*_*p*_ + 140 *− T*_*f*_ where *T*_*f*_ is a uniformly distributed random infection time between 0 and 84 days (i.e., during the first trimester).

Numerically simulating our model to the time of the first microcephaly detection *T*_*d*_ yields the prevalence of ZIKV in the import region at that time. The simulations are done using baseline parameter values as in Table 1, and with the random network distributions for sexually active males and females in the same region being Poisson with mean 2.25, and for males and females of different regions being Poisson with mean 0.1. These values are chosen so that the resulting basic reproduction number *R*_0_ and final size agree with literature values; see the Results section. We assume that the population of Region 1 is 2 million and that initially one person in Region 1 is infectious, while all other people and all mosquitoes in Regions 1 and 2 are susceptible.

The uncertainty in parameter values in Table 1 is incorporated in the simulations using the Latin Hypercube Sampling (LHS) maximin criteria [23], assuming all parameters except the death rate of mosquitoes *d* have uniform distributions with ranges given in Table 1, and *1/d* is normally distributed with mean *μ* = 50 days and standard deviation *σ* = 3 days. For estimating *T*_*p*_ and *T*_*d*_ distributions, we sample 2,000 points from the LH parameter space. For prevalence and total case estimates in the import region, we sample 100 points in the LH parameter space, and for each point selected, we compute the probability density function of the time until detection of ZIKV and sample a further 100 points from this distribution. We select LH data that gives rise to ZIKV epidemics with a least 1 pregnant female in a population of 2 million.

## Results

The basic reproduction number *R*_0_ is computed for the baseline parameters in Table 1: *R*_0_ ≈ 1*.*4 (TA, Additional file 1), agreeing with other estimated values [24]. For these parameters, the final sizes of the outbreaks in Regions 1 and 2 are numerically calculated to be 63.95% and 64.66%, respectively. These values are near the reported final sizes of outbreaks on Yap Island in 2007 and French Polynesia in 2013-2014.

**Fig. 2:**
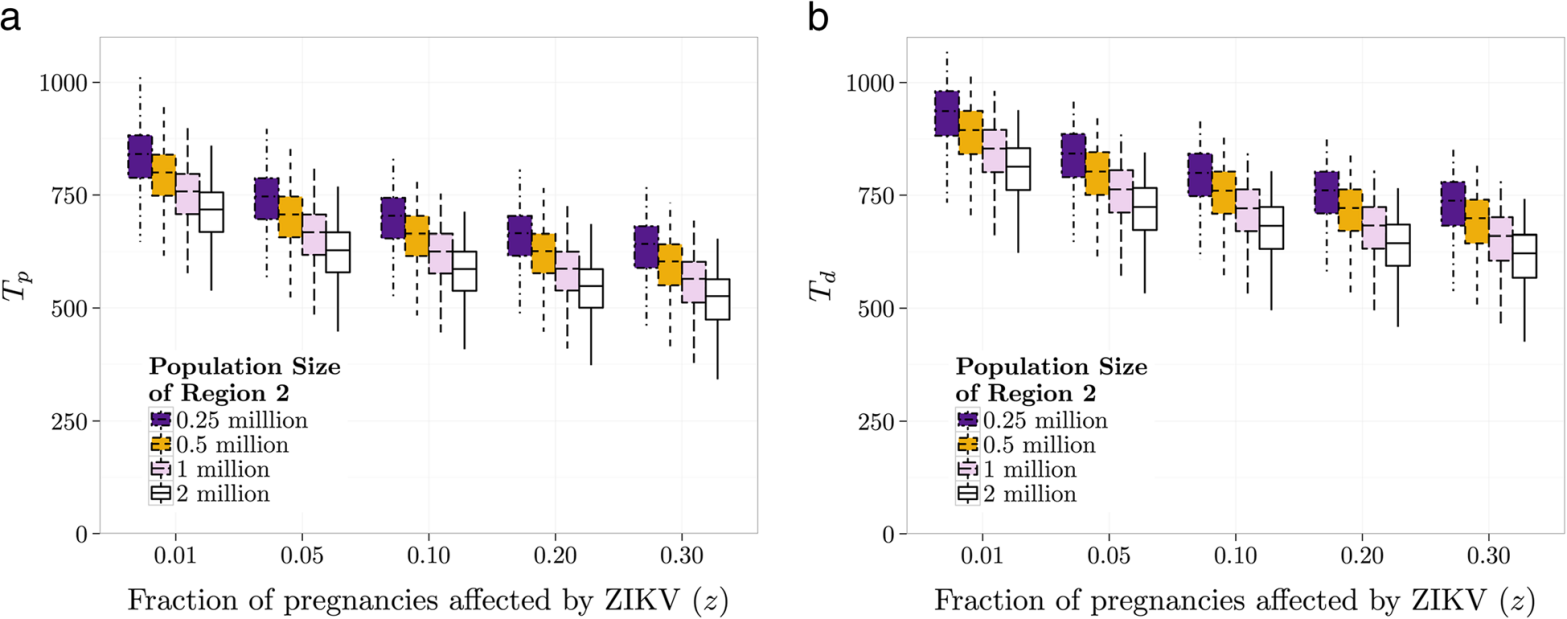
**a**. Time until the first pregnant female contracts ZIKV and passes it on to her fetus (*T*_*p*_). **b**. Time until detection of a ZIKV outbreak (*T*_*d*_). Here, *z* is the proportion of pregnancies resulting in abnormalities from ZIKV and Region 1 has a population of 2 million

For a range of *z* values, Fig. 2a shows box plots of the length of time until a pregnant female contracts ZIKV and passes it on to her fetus for population sizes in Region 2 ranging from 0.25 to 2 million. This median time lies between 525 and 800 days, depending on population size and *z* values. Figure 2b shows the box plots of the time to the first microcephaly detection for the same settings as used in Fig. 2a, giving a median time lying between 625 and 900 days. For every doubling of population size, both median times go down by at most 30 days.

**Fig. 3:**
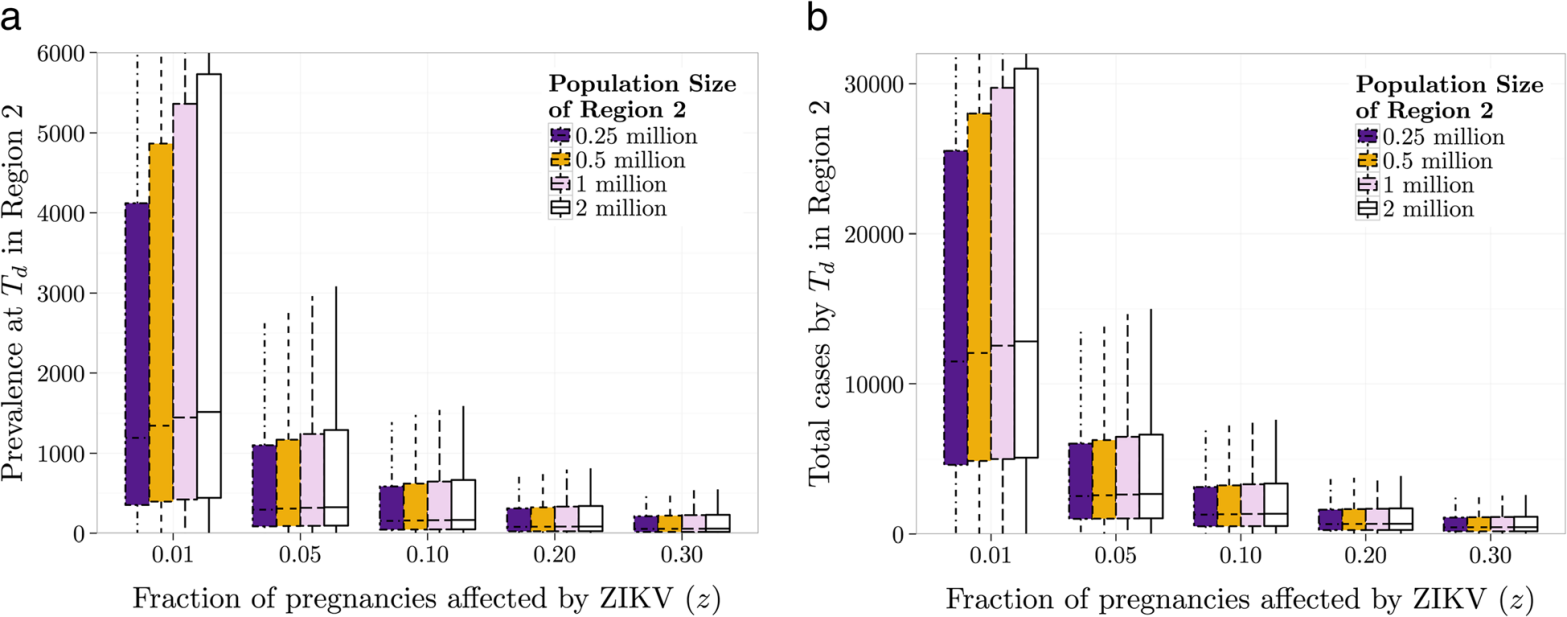
**a**. Prevalence of ZIKV at the time of detection as a function of the fraction of pregnancies that are affected by ZIKV. One hundred points are sampled (by LHS maximin criteria) from parameter values in Table 1 as well as *z*, assuming all parameters but *d* follow uniform distributions, and *d* ~ *N*(*μ* = 50,*σ* = 3). For every point, 100 subsequent points are selected randomly from the probability density function of the time until detection, and the prevalence at each of the 10,000 points is computed for each population size. These data points are summarized in boxplots. Note that the upper whisker of the boxplot for *z* = 0.01 with 2 million people extends to about 14,000 but is not shown in its entirety. For *z* = 0.01 with 0.25, 0.5 and 1 million people, and for *z* = 0.05 with 0.25 million people, we omit parameter sets in the LH for which microcephaly may not occur during an epidemic. **b**. Using the same sampling procedure as in **a**, total cases by the time of detection in the second region are determined as a function of *z*, and results are also presented in boxplots. Note that the upper whisker of the boxplot for *z* = 0.01 with 2 million people extends to about 70,000 but is not shown in its entirety. In both **a** and **b**, upper whiskers for all populations with *z* = 0.01 are not completely shown, and outliers are not plotted

Based on our sensitivity analysis procedure, we present in Fig. 3a boxplots of the 10,000 data points of prevalence (number of infectious individuals) at the time of detection *T*_*d*_ in the import region (Region 2) for fixed *z* values, and in Fig. 3b boxplots of 10,000 data points of the total cases by *T*_*d*_ in the import region for fixed *z* values. In Fig. 3a and b the populations of the import region are fixed at 0.25, 0.5, 1, and 2 million, whereas that of the source region remains at 2 million. Figure 3 shows that by the time a microcephaly case is detected in an import region with a population of at least 250,000, the median prevalence of ZIKV ranges from 200-1,500, and the median total infected ranges from 1,000-12,000. Larger population sizes do not significantly change these predictions.

## Discussion

In order to quantify ZIKV transmission from a source region to an import region, we formulated a mathematical model for the transmission of ZIKV in two regions. As in other ZIKV models [24, 25], we incorporate in our model vector transmission (between mosquitoes and humans), and sexual transmission (from sexually active males to females). We take account of population structure through a contact network for sexually active individuals, which is more realistic than the homogeneous mixing assumption of other models [24, 25]. Using data from the literature and estimated from travel, we obtain the probability distributions of the time to the detection of microcephaly *T*_*d*_ in the import region for various population sizes. Based on this, we compute the prevalence at detection of microcephaly in an import region. Our method for estimating *T*_*d*_ can be adapted to other models of diseases that cause microcephaly or other neurological disorders in a fetus, for example, rubella.

Our values of *T*_*d*_ presented in Fig. 3b agree with the recent identification of microcephaly in a region in Colombia around March 2016 [26] as it is likely that a single infectious individual was responsible for the spread of ZIKV from Asia to Brazil in the second half of 2013 [2], corresponding to a time delay of about 800 days from the arrival of ZIKV in the source region to the detection of fetal abnormalities in a fetus in the import region in Colombia. Based on the time until microcephaly was observed in Columbia, our results on *T*_*d*_ in Fig. 2a indicate it is more likely that the percentage of infected pregnancies is between 1% and 10%.

From our model estimates, upon detection of microcephaly in a region with a population of at least 250,000 people, on average 1,000–12,000 will have been infected, and on average 200–1,500 will be infectious at this time. Thus, it is imperative that potential import regions monitor for ZIKV infections at all times. If indeed 1% *≤ z ≤* 10%, total cases and prevalences are more likely to be on the higher end of the reported ranges, indicating even greater local dissemination of ZIKV, and a greater public health issue.

ZIKV may be also linked to Guillain-Barré Syndrome (GBS) and other neurological syndromes [27]. However, GBS may arise from many other causes [28]. These syndromes may have higher prevalences and shorter delays of onset than microcephaly. Thus our model suggests that monitoring for these syndromes seems to be beneficial for early detection of ZIKV, provided the link between ZIKV and GBS is established and quantified. Currently, detecting ZIKV by GBS cases may be less reliable than by microcephaly.

## Conclusions

Through simulations of our model, we have shown that by the first detection of a microcephaly case, or similar fetal abnormalities, a sizable fraction (between 0.4% and 4.8%) of the population will have been infected by ZIKV. This makes controlling of local ZIKV spread very difficult. It is thus important for public health agencies to adopt surveillance (and possibly quarantine and isolation) measures to travelers from a region with an ongoing epidemic to avoid severe threats of ZIKV importation.

## Additional file

Additional file 1: This file contains the main methods that are involved in the model formulation and the estimation of parameters. This file also contains the technical details relating to the computation of the basic reproduction number *R*_*0*_. (DOCX 139 kb)

## Abbreviations

GBS: Guillain-Barré Syndrome
TA: Technical Appendix (Additional file 1)
ZIKV: Zika virus
