# Supplementary material for: Estimation of Zika virus prevalence by appearance of microcephaly
Source: BMC Infect Dis. 2016 Dec 12;16:754. doi: 10.1186/s12879-016-2076-z (PMC5153823; doi:10.1186/s12879-016-2076-z)
Supplement: Additional file 1: — This file contains the main methods that are involved in the model formulation and the estimation of parameters. This file also contains the technical details relating to the computation of the basic reproduction number R 0. (DOCX 139 kb) [file 12879_2016_2076_MOESM1_ESM.docx]

# Technical Appendix

## Model Formulation

For each region *j* = 1*,* 2 where *j* = 1 is the source region and *j* = 2 is the import region, we keep track of only the female mosquito population *Vj* (as male mosquitoes do not bite humans); the human population is partitioned into sexually active males (between the ages of 15 and 64 years, denoted as subscript *Mj* ), sexually active females (between the ages of 15 and 64 years, denoted as subscript *Fj*), and sexually inactive humans (below 15 and over 64 years of age, denoted as subscript *Nj* ). This interval of ages for human sexual activity is consistent with data in the literature; see, for example, [1,2]. Within each of these groups, the population is further divided into fractions of susceptible (*S*), infected and infectious, and recovered individuals (*R*), with sexually active males having two infectious stages: one with the virus present in both blood and semen (*I*), and one with viral particles present only in semen (*J*).

For region *j*, we include mosquito (*V_j_* ) to human (*H_j_*), human to mosquito, and male to female ZIKV transmission, with $H_{j}=M_{j}+F_{j}+N_{j}$. A mosquito is assumed to bite a human randomly. We model the transmission of ZIKV in the mosquito population *V_j_* as a Susceptible-Infectious model (*2*), assume equal birth and death rates in the mosquito populations of both regions, and consider the fractions in each region, thus $S_{V_{j}}+I_{V_{j}}=1$. The incubation period for mosquitoes is neglected, but see [3- 5] where an incubating class is included in ZIKV models.

Sexual contacts between males and females are represented by a random bipartite directed network (*i.e.,* males and females are represented by the nodes, and their contacts are represented by arcs), because such contacts are in general not homogeneous in a region. The Miller-Volz formulation [6] is adapted to model the sexual contact network. For $i,j=1,2\boldsymbol{,}$ we denote *θ_ij_* as an arc from a male of region *i* to a female of region *j* that has not yet transmitted. Thus *θ_ii_* is an arc from a male to a female of the same region, and *θ_ij_* $(i\neq j)$ is an arc from a male to a female of the different region that has not yet transmitted. Similarly for $i,j=1,2,$ we denote *ϕij* as an arc from a male of region *i* to a female of region *j* that has not yet transmitted but with the male node infectious. For females of region *j*, *Pij* (*k*) denotes the probability distribution of having in-degree *k* from males of region *i*, and $\Psi ij (x) = \sum_{k=0}^{\infty} Pij(k)xk$ is the probability generating function of this probability distribution. Note that our model does not include female to male transmission or male to male transmission as such transmissions are thought to be rare, and certainly less common than male to female and vector transmission; see, for example, [7-9]. Vectors are modeled in a similar fashion as done by [10] for a contagious environment, *i.e.*, there is homogeneous mixing between mosquitoes and humans.

Human births and deaths are neglected during an epidemic. Thus all populations are constant and we take variables as fractions. According to the United Nations population data for 2015 [11], estimates of the United States population age structure are as follows: 34% of the population is below 15 or 65 and over years of age (nonsexually active); 33% are sexually active males between 15 and 65 years of age and 33% are sexually active females between 15 and 65 years of age. Thus the proportions in each of the three classes are taken to be the same, namely 1*/*3. Similar proportions are estimated for Brazil [11].

Figure 4. Flowchart of ZIKV transmission in Region *j* (*j*=*1,2*).

With parameters defined as in the Table (in the main text), our model is based on the flowcharts presented in Figure 1 (in the main text) and Figure 4 and is formulated for *i, j* = 1*,* 2 as

$$\frac{dS_{M_{j}}}{dt}=-\frac{S_{M_{j}}}{3}\sum_{\mathcal{l=}1}^{2} \beta_{V_{\mathcal{l}}H_{j}}I_{V_{\mathcal{l}}}$$

$$\frac{dI_{M_{j}}}{dt}=\frac{S_{M_{j}}}{3}\sum_{\mathcal{l=}1}^{2} \beta_{V_{\mathcal{l}}H_{j}}I_{V_{\mathcal{l}}}-\gamma_{B}I_{M_{j}}$$

$$\frac{dJ_{M_{j}}}{dt}=\gamma_{B}I_{M_{j}}-\gamma_{S}J_{M_{j}}$$

$$\frac{dS_{F_{j}}}{dt}=-S_{F_{j}}\sum_{\mathcal{l=}1}^{2} \left( \beta_{M_{\mathcal{l}}F_{j}}\phi_{\mathcal{l}j}\frac{\Psi_{\mathcal{l}j}^{'}( \theta_{\mathcal{l}j})}{\Psi_{\mathcal{l}j}( \theta_{\mathcal{l}j})}+\frac{\beta_{V_{\mathcal{l}}H_{j}}}{3}I_{V_{\mathcal{l}}} \right)$$

$$\frac{dI_{F_{j}}}{dt}=S_{F_{j}}\sum_{\mathcal{l=}1}^{2} \left( \beta_{M_{\mathcal{l}}F_{j}}\phi_{\mathcal{l}j}\frac{\Psi_{\mathcal{l}j}^{'}( \theta_{\mathcal{l}j})}{\Psi_{\mathcal{l}j}( \theta_{\mathcal{l}j})}+\frac{\beta_{V_{\mathcal{l}}H_{j}}}{3}I_{V_{\mathcal{l}}} \right)-\gamma_{B}I_{F_{j}}$$

$$\frac{dI_{V_{j}}}{dt}=(1-I_{V_{j}}) \sum_{\mathcal{l=}1}^{2} \left( \beta_{H_{\mathcal{l}}V_{j}}\frac{I_{F_{\mathcal{l}}}}{3}+\frac{I_{M_{\mathcal{l}}}}{3}+\frac{I_{N_{\mathcal{l}}}}{3} \right)-dI_{V_{j}}$$

$$\frac{dS_{N_{j}}}{dt}=-\frac{S_{N_{j}}}{3}\sum_{\mathcal{l=}1}^{2} \beta_{V_{\mathcal{l}}H_{j}}I_{V_{\mathcal{l}}}$$

$$\frac{dI_{N_{j}}}{dt}=\frac{S_{N_{j}}}{3}\sum_{\mathcal{l=}1}^{2} \beta_{V_{\mathcal{l}}H_{j}}I_{V_{\mathcal{l}}}-\gamma_{B}I_{N_{j}}$$

$$\frac{d\theta_{ij}}{dt}=-\beta_{M_{i}F_{j}}\phi_{ij}$$

$$\frac{d\phi_{ij}}{dt}=-\beta_{M_{i}F_{j}}\phi_{ij}-({\gamma_{B}^{-1}+\gamma_{S}^{-1})}^{-1}\phi_{ij}+\frac{S_{M_{i}}}{3}\sum_{\mathcal{l=}1}^{2} \beta_{V_{\mathcal{l}}H_{i}}I_{V_{\mathcal{l}}}$$

## Parameter Values

Since people recover from the disease within a few days to 11 days [12] and the virus is not found in the blood after this time, we take $\frac{1}{\gamma_{B}}=7$ days. There has been evidence that a male carried the ZIKV and was still potentially infectious after 62 days of the initial infection [13]. Further studies show that the virus could be cultured 69 days after infection [14]*.* Since we consider an average value, we take $\frac{1}{\gamma_{S}}=55$ days, thus $\frac{1}{\gamma_{S}}+\frac{1}{\gamma_{B}}=62$days is the average time until ZIKV is not infectious from semen. We estimate $\beta_{M_{i}F_{i}}=0.5$, *i.e.,* the probability of transmission per day of males to females of the same region is 1/2. For males and females of different regions, we estimate the probability of transmission per day to be$\beta_{M_{i}F_{j}}=0.01$ for $i\neq j$, since the contact between a female of one region and a male of the other region is much smaller than for individuals of the same region. In [15], the mosquito biting rate for *Aedes aegypti* is estimated to be between 0.33 and 1 day^-1^ with the baseline value set at 0.5 day^-1^, and the mosquito biting rate for *Aedes albopictus* is estimated to be between 0.19 day^-1^ and 0.39 day^-1^, with the baseline value set at 0.26 day^-1^. Since ZIKV is thought to be transmitted by both of these species of mosquitoes, we take the biting rate of mosquitoes to be 0.35 day^-1^. We take the probability of transmission of infection in one bite from mosquito to human and from human to mosquito to both be 0*.*3 for individuals and mosquitoes in the same region, which is about the midpoint of the values presented in [15] for dengue and chikungunya; thus $\beta_{V_{i}H_{i}}=\beta_{H_{i}V_{i}}=0.105$. For mosquito to human and human to mosquito transmission from region *i* to region *j*, we estimate that every day, 0*.*002% of the population of region *i* visits region *j*. According to data from 2011 [16], about 120*,*000 Brazilians visited the United States each month; with an approximate population of 200 million people in Brazil, this represents 0*.*002% of the total population entering the United States every day. For United States to foreign travel, 0*.*002% of the population represents 126,000 Americans visiting a foreign city in a span of three weeks. Assuming that each visitor from region *i* remains in region $j\neq i$ for an average of 14 days, $\beta_{V_{i}H_{j}}=\beta_{H_{i}V_{j}}=0.00002 \times14 \times0.105=0.00003$day^-1^, for $i\neq j$.

## Basic Reproduction Number

To compute the basic reproduction number, we use the next-generation matrix method [17] with infected classes *IV*1 , *IV*2 , *IF*1 , *IF*2 , *IM*1 , *IM*2 , *JM*1 , *JM*2 , *IN*1 , *IN*2 , *φ*11, *φ*12, *φ*21 and *φ*22. Thus the Jacobian *F − V* at the disease free equilibrium has

$$F=\left( \begin{matrix} O & B_{1} & B_{1} & O & B_{1} & O & O \\ B_{2} & O & O & O & O & B_{3a} & B_{3b} \\ B_{2} & O & O & O & O & O & O \\ O & O & O & O & O & O & O \\ B_{2} & O & O & O & O & O & O \\ B_{4a} & O & O & O & O & O & O \\ B_{4b} & O & O & O & O & O & O \end{matrix} \right), V=\left( \begin{matrix} D & O & O & O & O & O & O \\ O & G_{b} & O & O & O & O & O \\ O & O & G_{b} & O & O & O & O \\ O & O & -G_{b} & G_{s} & O & O & O \\ O & O & O & O & G_{b} & O & O \\ O & O & O & O & O & A_{1} & O \\ O & O & O & O & O & O & A_{2} \end{matrix} \right)$$

where the 2-by-2 matrices in *F* and *V* are given by

$$B_{1}=\frac{1}{3}\left( \begin{matrix} \beta_{H_{1}V_{1}} & \beta_{H_{2}V_{1}} \\ \beta_{H_{1}V_{2}} & \beta_{H_{2}V_{2}} \end{matrix} \right), B_{2}=\frac{1}{3}\left( \begin{matrix} \beta_{V_{1}H_{1}} & \beta_{V_{2}H_{1}} \\ \beta_{V_{1}H_{2}} & \beta_{V_{2}H_{2}} \end{matrix} \right),$$

$$B_{3a}=\left( \begin{matrix} {\beta_{M_{1}F_{1}}\Psi}_{11}^{'}\left( 1 \right) & 0 \\ 0 & {\beta_{M_{1}F_{2}}\Psi}_{12}^{'}\left( 1 \right) \end{matrix} \right),$$

$B_{3b}=\left( \begin{matrix} {\beta_{M_{2}F_{1}}\Psi}_{21}^{'}\left( 1 \right) & 0 \\ 0 & {\beta_{M_{2}F_{2}}\Psi}_{22}^{'}\left( 1 \right) \end{matrix} \right),$ $B_{4a}=\frac{1}{3}\left( \begin{matrix} \beta_{V_{1}H_{1}} & \beta_{V_{2}H_{1}} \\ \beta_{V_{1}H_{1}} & \beta_{V_{2}H_{1}} \end{matrix} \right),$

$$B_{4b}=\frac{1}{3}\left( \begin{matrix} \beta_{V_{1}H_{2}} & \beta_{V_{2}H_{2}} \\ \beta_{V_{1}H_{2}} & \beta_{V_{2}H_{2}} \end{matrix} \right),$$

$$D=diag\left\{ d,d \right\}, G_{b}=diag\left\{ \gamma_{B},\gamma_{B} \right\}, G_{s}=diag\left\{ \gamma_{S},\gamma_{S} \right\},$$

$A_{i}=diag\left\{ \beta_{M_{i}F_{1}}+({\gamma_{B}^{-1}+\gamma_{S}^{-1})}^{-1},\beta_{M_{i}F_{2}}+({\gamma_{B}^{-1}+\gamma_{S}^{-1})}^{-1} \right\}$ for $i=1,2,$

and *O* denotes the 2 *×* 2 zero matrix.

Hence it follows that

$$V^{-1}=\left( \begin{matrix} D^{-1} & O & O & O & O & O & O \\ O & {G_{b}}^{-1} & O & O & O & O & O \\ O & O & {G_{b}}^{-1} & O & O & O & O \\ O & O & {G_{s}}^{-1} & {G_{s}}^{-1} & O & O & O \\ O & O & O & O & {G_{b}}^{-1} & O & O \\ O & O & O & O & O & {A_{1}}^{-1} & O \\ O & O & O & O & O & O & {A_{2}}^{-1} \end{matrix} \right)$$

and thus,

$${R_{0}=\rho(FV}^{-1})=\rho\left( \begin{matrix} O & B_{1}{G_{b}}^{-1} & B_{1}{G_{b}}^{-1} & O & B_{1}{G_{b}}^{-1} & O & O \\ B_{2}D^{-1} & O & O & O & O & B_{3a}{A_{1}}^{-1} & B_{3b}{A_{2}}^{-1} \\ B_{2}D^{-1} & O & O & O & O & O & O \\ O & O & O & O & O & O & O \\ B_{2}D^{-1} & O & O & O & O & O & O \\ B_{4a}D^{-1} & O & O & O & O & O & O \\ B_{4b}D^{-1} & O & O & O & O & O & O \end{matrix} \right),$$

where *ρ* denotes the spectral radius. Numerical calculation for our baseline parameter values gives

*R*_0_ *≈* 1*.*4.

# References

1. Lindau ST, Schumm LP, Laumann EO, Levinson W, O’Muircheartaigh CA, Waite LJ. A study of sexuality and health among older adults in the United States. N Eng J Med 2007;357:762–774.
2. Weinstock H, Berman S, Cates W. Sexually transmitted diseases among American youth: incidence and prevalence estimates, 2000. Perspect Sex Reprod Health 2004;36: 6–10.
3. Kucharski AJ, Funk S, Eggo RM, Mallet HP, Edmunds WJ, Nilles EJ. Transmission dynamics of Zika virus in island populations: A modelling analysis of the 2013-14 French Polynesia outbreak. PLoS Negl Trop Dis 2016;10:1–15.
4. Towers S, Brauer F, Castillo-Chavez C, Falconar A, Mubayi A, Romero-Vivas C. Estimation of the reproduction number of the 2015 Zika virus outbreak in Barranquilla, Colombia, and a first estimate of the relative role of sexual transmission. arXiv 2016; 1606.01422v1:Online.
5. Gao D, Lou Y, He D, Porco TC, Kuang Y, Chowell G, et al. Prevention and control of Zika as a mosquito-borne and sexually transmitted disease: A mathematical modeling analysis. Sci Rep 2016;10.1038/srep28070:Published online before print.
6. Miller J. A note on a paper by Erik Volz: SIR dynamics in random networks. J Math Biol 2011;62:349–358.
7. Davidson A, Slavinski S, Komoto K, Rakeman J, Weiss D. Suspected female-to-male sexual transmission of Zika virus-New York City, 2016. MMWR Morb Mortal Wkly Rep 2016;65:716-717.
8. Deckard, DT, Chung WM, Brooks JT, Smith JC, Woldai S, Hennessey M, et al. Male-to-male sexual transmission of Zika virus-Texas, January 2016. MMWR Morb Mortal Wkly Rep 2016;65:372-374.
9. Purcell DW, Johnson CH, Lansky A, Prejean J, Stein R, Denning P, et al. Estimating the population size of men who have sex with men in the United States to obtain HIV and syphilis rates. Open AIDS J 2012;6:98-107.
10. Li M, Ma J, van den Driessche P. Model for disease dynamics of a waterborne pathogen on a random network. J Math Biol 2015;72:961–977.
11. World Population Prospects: The 2015 Revision, Volume I: Comprehensive Tables. Tech- nical Report ST/ESA/SER.A/379, United Nations, Department of Economic and Social Affairs, Population Division, 2015.
12. Petersen EE, Polen K, Meaney-Delman D, Ellington S, Oduyebo T, Cohn A, et al. Update: interim guidance for health care providers caring for women of reproductive age with possible Zika Virus exposure-United States, 2016. MMWR Morb Mortal Wkly Rep 2016;65:315–322.
13. Atkinson B, Hearn P, Afrough B, Lumley S, Carter D, Aarons E, et al. Detection of Zika virus in semen. Emerg Infect Dis 2016;22:940.
14. Petersen, EE, Meaney-Delman D, Nebett-Fanfair R, Havers F, Oduyebo T, Hills SL, et al. Update: interim guidance for preconception counseling and prevention of sexual transmission of Zika virus for persons with possible Zika virus exposure — United States, September 2016. MMWR Morb Mortal Wkly Rep 2016;65:1077–1081.
15. Manore C, Hickmann K, Xu S, Hyman JM. Comparing dengue and chikungunya emergence and endemic transmission in *A. aegypti* and *A. albopictus*. J Theor Biol 2014;356:174–191.
16. Riker D, Vila-Goulding J. The boom in Brazilians traveling to the United States. Journal of International Commerce & Economics 2013;5:1–15.
17. van den Driessche P, Watmough J. Reproduction numbers and sub-threshold endemic equilibria for compartmental models of disease transmission. Math Biosci 2002;180:29– 48.
